# One health approach to study human health risks associated with *Dermanyssus gallinae* mites

**DOI:** 10.1016/j.heliyon.2024.e30539

**Published:** 2024-05-01

**Authors:** Pavle Banović, Angélique Foucault-Simonin, Luka Papić, Sara Savić, Aleksandar Potkonjak, Aleksandar Jurišić, Marko Radenković, Dragana Mijatović, Verica Simin, Ivana Bogdan, Zbigniew Zając, Joanna Kulisz, Aneta Woźniak, David Hartmann, Jan Perner, Alejandra Wu-Chuang, Lourdes Mateos-Hernandez, Sara Moutailler, Alejandro Cabezas-Cruz

**Affiliations:** a Clinic for Lyme Borreliosis and Other Tick-Borne Diseases, Department of Prevention of Rabies and Other Infectious Diseases, Pasteur Institute Novi Sad, Novi Sad, 21000, Serbia; b Department of Microbiology with Parasitology and Immunology, Faculty of Medicine, University of Novi Sad, Novi Sad, 21000, Serbia; c Diagnostics and Laboratory Research Task Force, Balkan Association for Vector-Borne Diseases, 21000, Novi Sad, Serbia; d ANSES, INRAE, Ecole Nationale Vétérinaire d’Alfort, UMR BIPAR, Laboratoire de Santé Animale, Maisons-Alfort, France; e Veterinary clinic “Darvin”, Bate Brkića 32, Novi Sad, 21000, Serbia; f Scientific Veterinary Institute “Novi Sad”, 21000, Novi Sad, Serbia; g Department of Veterinary Medicine, Faculty of Agriculture, University of Novi Sad, 21000, Novi Sad, Serbia; h Department for Environmental and Plant Protection, Faculty of Agriculture, University of Novi Sad, Novi Sad, Serbia; i Department of Biology and Ecology, Faculty of Sciences, University of Novi Sad, Trg Dositeja Obradovića 3, 21000, Novi Sad, Serbia; j Department for Research & Monitoring of Rabies & Other Zoonoses, Pasteur Institute Novi Sad, 21000, Novi Sad, Serbia; k Department of Microbiology, Pasteur Institute Novi Sad, 21000, Novi Sad, Serbia; l Department of Biology and Parasitology, Medical University of Lublin, Radziwiłłowska 11, 20-080, Lublin, Poland; m Institute of Parasitology, Biology Centre, Czech Academy of Sciences, 37005, Ceske Budejovice, Czech Republic

**Keywords:** Red mites, Allergy, One health, Pathogens

## Abstract

Despite the significant health risks associated with *Dermanyssus gallinae* infestations in humans, they are often overlooked. This study investigated a household case of *D. gallinae* infestation and explored the resulting clinical manifestations and risk of infection in family members. Microfluidic PCR was employed for high-throughput screening of pathogens in collected mites and blood samples from both chickens and family members. Morphological and molecular examinations confirmed the identity of the mites as *D. gallinae* sensu stricto (s.s.), with evidence indicating recent blood feeding. Results indicated that the mites exclusively harbored various pathogens, including *Bartonella* spp., *Ehrlichia* spp., Apicomplexa, and *Theileria* spp. Blood samples from family members and poultry tested negative for these pathogens, suggesting a potential reservoir role for *D. gallinae*. The study further identified haplotypes of *D. gallinae*, classifying them into *D. gallinae* s.s., cosmopolitan haplogroup A. Serological analysis revealed elevated IgE seroreactivity against mite proteins in the family member with bite lesions. Antibodies against *Bartonella* spp. were detected in this individual, indicating exposure to the pathogen. In summary, this study sheds light on the clinical manifestations, pathogen detection, and genetic characterization of *D. gallinae* infestations, underscoring the necessity of adopting comprehensive approaches to manage such infestations effectively.

## 1 Introduction

The red-poultry mite, *Dermanyssus gallinae* (Arthropoda: Dermanyssidae) is a blood-sucking ectoparasite primarily of birds and is widely regarded as a poultry pest and poses a significant threat to the laying-hen industry worldwide [1]. These mites can be considered micro-predators, as suggested by recent research [2] and are known to infest poultry in farms or yards. Rapid multiplication of this organism can lead to adverse effects such as anemia, malnutrition, and a decline in egg quality and quantity [[2], [3], [4]]. In severe cases, it may even result in the death of the host. Depending on its plasticity, *D. gallinae* can feed on non-avian hosts, such as mammal pets, horses, goats, and humans [5].

*Dermanyssus gallinae* is often considered a vector for several bacterial pathogens, such as *Salmonella* Gallinarum, *Salmonella* Enteritidis, *Pasteurella multocida*, *Coxiella burnetii*, and Spirochetes, as well as viral pathogens including Western equine encephalitis virus, and Venezuelan equine encephalitis virus [[6], [7], [8]]. Additionally, the poultry red mite has been found to test positive for various tick-borne pathogens (TBPs), although its ability to transmit them to other hosts has not been conclusively demonstrated [4]. Specifically, there are reports of *D. gallinae* as an occasional vector for *Borrelia bugdorferi*, the causative agent of Lyme disease [[9], [10], [11]] transmitted mainly by ticks [12].

Additionally, *D. gallinae* have been implicated as a potential vector and reservoir for *Erysipelothrix rhusiopathiae*, the pathogen responsible for swine erysipelas [13]. In the case of Eastern equine encephalitis (EEE), studies by Clark et al. [14] suggested that other dermanyssoid mites such as *Haemogamasus liponyssoides* and *Ornithonyssus bacoti* can ingest the virus while feeding on infected mice, though no viral replication or transmission through bites were observed. On the other hand, *D. gallinae*, when fed on infected chicks, can remain carriers for at least a month without viral replication, transmitting the virus to other chicks through bites during blood meals [15,16]. For Western equine encephalitis (WEE), viral strains were isolated from *Ornithonyssus sylviarum* and *Dermanyssus americanus*, suggesting a potential role in transmission [17,18]. In the context of Venezuelan equine encephalitis virus (VEE), *D. gallinae* was identified as a mechanical vector in transmission experiments, but without viral replication or transovarian transmission [16].

Concerning *Coxiella burnetii*, *D. gallinae* was shown to transmit the bacteria between guinea pigs and birds during blood meals, highlighting its role as a vector of this bacterium [19]. Additionally, *Allodermanyssus sanguineus* is recognized as a principal vector of *Rickettsia akari* among the Dermanyssoidea for bacteria of the *Rickettsia* genus, while other species within this superfamily still require further study to confirm their vectorial roles [19]. Furthermore, high-throughput sequencing revealed that *Bartonella* bacteria constituted a substantial portion of the mite microbiome, suggesting their role as potential vectors [20,21]. Indeed, a prior study documented an outbreak of *Bartonella quintana* infection in a young family of high socioeconomic status and their visiting relatives, situated in a regional city in northern Czech Republic [22]. The epidemiological significance of these findings in the field remains to be fully defined for both viruses causing equine encephalitis and bacteria of the genera *Rickettsia*, *Bartonella* and *Borrelia*.

While *D. gallinae* is primarily associated with avian hosts, there have been reports suggesting a shift towards generalism in host choice [23]. Factors such as environmental changes, globalization, transportation, and increasing human and animal population densities have contributed to more frequent encounters, thereby stimulating their expansion to different hosts [23]. In recognition of its substantial impact on the well-being of both humans and animals, *D. gallinae* is acknowledged as an emerging threat by international organizations such as the Food and Agriculture Organization, the World Organization for Animal Health, and the World Health Organization [24]. Consequently, managing this pest falls under the purview of the One Health approach, which emphasizes a multidisciplinary and collaborative approach to address health issues [[24], [25], [26]].

In humans, bites from *D. gallinae* can provoke dermatitis, and similar reactions can be observed in dogs and cats, including symptoms like pruritus and skin lesions [5,27,28]. Human infestations with *D. gallinae* can occur as an occupational hazard, particularly among poultry farm workers, or through accidental exposure in urban environments [24]. While it has been confirmed that *D. gallinae* injects its DNA into the host's skin during blood meals, there is limited data available on its ability to transmit pathogens to human hosts [29]. The mites' nocturnal biting activity and persistence can significantly reduce the quality of life for infested individuals, particularly when the cause of the presented lesions is not timely identified [[30], [31], [32], [33]].

This paper aims to comprehensively investigate the threat posed by the red-poultry mite, *D. gallinae*, and its potential role as a vector for various pathogens in both poultry and humans. Recognizing its significant impact on the laying-hen industry and its emergence as a threat to human and animal health, the study adopts a One Health approach. Focusing on a household infestation in Serbia, the research integrates medical observations, molecular diagnostics, and serological analyses to assess pathogen circulation in areas infested with *D. gallinae*. By employing high-throughput sequencing, phylogenetic analysis, and microfluidic real-time PCR, the study aims to shed light on the diversity of pathogens carried by the mites and their potential transmission to humans and poultry. Additionally, the investigation delves into the morphological identification of *D. gallinae*, its feeding behavior, and the potential role of this mite as a vector for bacterial and viral pathogens. Through a multidisciplinary approach, this paper strives to contribute valuable insights into the epidemiological significance of *D. gallinae*, emphasizing its impact on both the poultry industry and public health.

## 2 Materials and methods

### 2.1 Household mite infestation in family members and study design

#### 2.1.1 Precedents of *D. gallinae* infestation in the household

On June 14th, 2022, a family residing in Petlovo Brdo, an urban neighborhood of in Belgrade (coordinates: 44.7200°N, 20.4231°E) visited the Pasteur Institute Novi Sad to report a suspected tick infestation in their recently constructed two-story house. The household comprised three family members: the father (Patient 1), mother (Patient 2), and son (Patient 3). Patient 1, a 52-year-old man with no prior medical conditions, experienced an attack the previous night (June 13th), believing it to be caused by "ticks". Intense itching and pain prompted him to remove his shirt, which was then placed in a plastic bag outside.

**Fig. 1:**
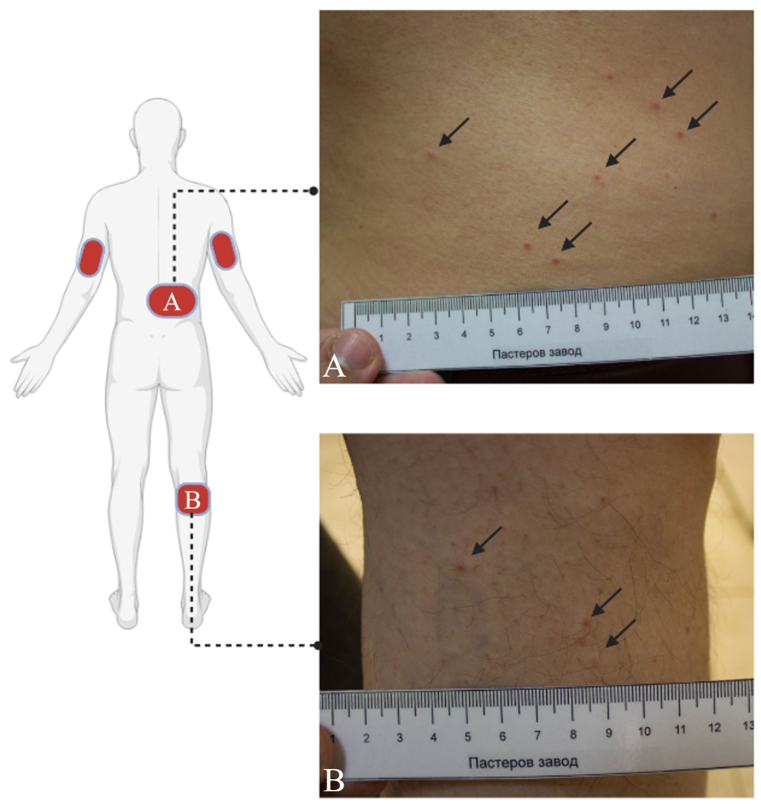
**Location of lesions caused by *Dermanyssus gallinae* in the patient.** (A) On the posterior side of the lower leg, look for the black arrows to find the biting sites. (B) On the lateral side of the abdomen, locate the biting sites using the black arrows. Red patches show the distribution of bite lesions. Created with BioRender.com. (For interpretation of the references to colour in this figure legend, the reader is referred to the Web version of this article.)

During the examination, Patient 1 complained of a severe itching at the bite sites. A subsequent physical examination on Patient 1, revealed hives-like skin lesions on the abdomen (Fig. 1A), legs (Fig. 1B), and arms. Scratching marks and papules were evident only on his skin. No other family members reported previous tick-borne diseases (TBDs). Seroconversion testing for *D. gallinae* pathogens was initiated with a serum sample from Patient 1. An antihistamine drug (Loratadine) was prescribed, and a follow-up examination was scheduled in 7 days, with instructions to report any new signs or symptoms.

Capillary and venous blood samples were collected from skin lesions of Patient 1 and all family members, respectively, to identify bacteria and parasites associated with TBDs. Capillary blood samples were obtained by using a lancet, as described previously [[34], [35], [36]] [[34], [35], [36]] [[34], [35], [36]]. Considering the potential for any of the family members to be in the incubation period and possibly develop conditions requiring further medical attention, serum samples were also collected. This facilitated serology examinations through paired serum samples if signs of disease emerged within the subsequent four weeks.

The Patient 1 was instructed to bring the previously packed shirt to the Pasteur Institute Novi Sad for entomological examination. On June 15th, it was determined that mites, not ticks, were responsible for the infestation. The mites were grouped into four pools for DNA extraction and subsequent molecular analysis, involving pathogen detection through microfluidic real-time PCR and conventional PCR, as well as molecular identification of the mites.

As none of the family members exhibited signs of disease in the subsequent four weeks, only Patient 1, with prior skin lesions, was requested for a second examination and blood sample collection. No visible lesions were observed, and antihistamine treatment was discontinued during the second examination. No signs of acute infection were noted. The second blood sample from Patient 1 would be analysed for exposure to vector-associated pathogens using microfluidic real-time PCR. Human serum samples were screened for *Bartonella* spp. and *Ehrlichia* spp. exposure and *D. gallinae*-specific IgE antibodies.

#### 2.1.2 Study design using a one health approach

In addressing the suspected infestation of zoonotic bloodsucking mites, a study was undertaken employing a One Health approach to assess their presence and associated pathogens within a household situated on Petlovo Brdo. On June 16th, a field trip was organized to inspect the household for mites and potential sources. The poultry owned by the family, comprising three chickens and one turkey situated in the chicken coop located approximately 25 m from the house, underwent thorough examination for mites, with blood samples collected.

Each bird was individually captured and restrained to facilitate a thorough examination of their feathers and skin for the presence of mites, including the chicken coop area. Throughout our inspection, we did not find any mites in the household buildings or the chicken coop, which was examined by a veterinarian. Chicken coop was examined via inspection of dust and feathers taken from the chicken coop floor, as described previously [37].

Simultaneously, the household and yard were inspected to identify nests of synanthropic or wild birds. To assess the presence of pathogens, microfluidic real-time PCR (see below section 2.4.3 Microfluidic real-time PCR) was employed to analyze blood and platelet samples. Additionally, mites specimens extracted from Patient 1 clothing were also subjected to analysis.

#### 2.1.3 Household description

The household of interest consisted of one central residential building and two smaller structures used as a garage and shed. Additionally, there was a spacious terrace and a backyard spanning approximately 200 square meters. Within the backyard, a small fenced chicken coop was present, situated beside a field overgrown with acacia and other trees. During our visit, we observed various bird species landing on the terrace, including common woodpigeons (*Columba palumbus*) and magpies (*Pica pica*). Crows and woodpeckers were seen in the part of the backyard with dense tree growth, as some of these trees bore fruit and served as a food source for the birds. Furthermore, we discovered several vacant nests near the path commonly used by household members when traversing the overgrown area of the backyard.

### 2.2 Morphological identification of *D. gallinae* and estimation of feeding period

#### 2.2.1 Morphological identification of *D. gallinae* using Scanning Electron Microscope

All mites were carefully removed from the patients' shirts using tweezers. The mites were then placed in 70 % ethanol and morphologically identified using a Scanning Electron Microscope (SEM; JEOL JSM 6460 LV, Tokyo, Japan) and standard taxonomic keys [38,39].

Before observation under SEM, mites were placed on holders with conductive double sided carbon tape and ion sputter coated with gold using Bal-Tec SCD 005 Sputter Coater over 90 s at a working distance of 50 mm at 30 mA. Images were acquired with the SEM operating at 20 kV in high vacuum.

#### 2.2.2 Estimation of *D. gallinae* feeding period

To correlate the anamnestic data provided by the patients with feeding time of *D. gallinae*, were employed morphological keys outlined by Ma et al. [3]. This determination was executed using a stereomicroscope (SLX-1, Optika S. r.l., Ponteranica, Italy) and a digestion score system. A score of 0 signified that the mites had just finished feeding, characterized by a red body full of blood. Score 1 indicated a day of digestion, with the trunk of the mite predominantly red and small transparent areas. Score 2 corresponded to a two-day digestion period, with observable blood in three pairs of caeca and the hindgut, while the central parts of the hind body were transparent. Score 3 denoted a period of 3–5 days of digestion, with the body of the mite mostly transparent and only a small amount of blood visible in the intestine. Finally, score 4 represented the 6th day after a blood meal, where the body of the mite was almost completely transparent or white.

### 2.3 Serum separation and platelet extraction

Blood samples from humans and animals were collected using BD Vacutainer® SST™ Tubes (BD, Franklin Lakes, NJ, United States) and allowed to clot at room temperature. Subsequently, the samples were centrifuged at 2000×*g* for 10 min to obtain serum. The serum was then extracted and stored at −80 °C until further use in serological analyses for the research of IgM and IgG antibodies reactive with *Bartonella* spp. and *Ehrlichia* spp. (refer to section 2.7.1 below titled ‘Testing for IgM and IgG antibodies reactive with *Bartonella* spp. and *Ehrlichia* spp. in human serum samples’).

To extract platelets from the whole blood, the method described by Sanderson et al. [40] was employed. Platelet-rich plasma was separated from the whole blood by centrifugation at 120*g* for 20 min. Afterward, an additional centrifugation step at 400 g for 20 min was performed to separate the platelets from the platelet-rich plasma. The successful separation of the platelet fraction from each patient's blood sample was confirmed by observing white sediment. The platelet fractions were individually placed in sterile Eppendorf vials and stored at −80 °C until DNA extraction.

### 2.4 Molecular analysis of blood and mite samples

#### 2.4.1 Nucleic acid extraction

Complete blood, capillary blood, and platelet DNA were isolated using the Nucleospin Tissue kit (Macherey Nagel, Düren, Germany), following the manufacturer's instructions. To prepare the samples, we homogenized four pools of mites, ensuring they were at the same stages and feeding status. Each pool comprised approximately 24 mites. This homogenization process involved using 180 μL of Lysis buffer (T1 buffer) and 25 μL of proteinase K from the Nucleospin Tissue kit (Macherey Nagel). The homogenization was carried out with a Precellys 24 lyser/homogenizer (Bertin Technologies, Montigny-le-Bretonneux, France) equipped with 2.8 mm stainless steel beads, operating at a speed of 3724×*g* for 20 s. The homogenates were then incubated for 3 h at 56 °C, and DNA extraction was carried out following the manufacturer's instructions. Finally, the purified DNA was eluted in 50 μL of elution buffer.

#### 2.4.2 DNA pre-amplification for microfluidic real-time PCR

To enhance the detection of pathogen DNA, the total DNA was pre-amplified using the PreAmp Master Mix (Fluidigm, San Francisco, CA, USA), following the manufacturer's instructions. Primers (Supplementary Table S1), which targeted all pathogens, were pooled by combining an equal volume of each primer, resulting in a final concentration of 200 nM for each primer. The reaction was conducted in a final volume of 5 μL, comprising 1 μL of PreAmp Master Mix, 1.25 μL of the pooled primer mix, 1.5 μL of distilled water, and 1.25 μL of DNA. The thermocycling program consisted of one cycle at 95 °C for 2 min, followed by 14 cycles at 95 °C for 15 s and 60 °C for 4 min. After completing the cycling program, the reactions were diluted 1:10 in Milli-Q ultrapure water. The pre-amplified DNAs were stored at −20 °C until required.

#### 2.4.3 Microfluidic real-time PCR

To identify major TBPs (25 bacterial species, 7 parasite species, 5 bacterial genera, 3 parasite genera) (Supplementary Table S1), we utilized the BioMark™ real-time PCR system from Fluidigm (San Francisco, CA, USA). This system facilitated high-throughput microfluidic real-time PCR amplification using 48.48 Dynamic Array™ IFC chips (Fluidigm, San Francisco, CA, USA). These chips allowed for the dispensing of 48 PCR mixes and 48 samples into individual wells. On-chip microfluidics then assembled real-time PCR reactions in separate chambers before thermal cycling, resulting in 2304 individual reactions.

In summary, the amplification process involved the use of 6-carboxyfluorescein (FAM)- and black hole quencher (BHQ1)-labeled TaqMan probes with PerfeCTa® qPCR ToughMix®, Low ROX™ (QuantaBio, Beverly, MA, USA), following the manufacturer's instructions. The PCR cycling consisted of an initial step of 2 min at 50 °C, followed by 10 min at 95 °C. Subsequently, 40 cycles of two-step amplification were performed, with each cycle consisting of 15 s at 95 °C and 1 min at 60 °C. To serve as a negative control, one water control was included per chip.

To assess whether the sample contained any inhibitory factors that could interfere with the PCR, we added *Escherichia coli* strain EDL933 DNA to each sample as an internal inhibition control. Specific primers and a probe for the *E. coli eae* gene were used for this purpose.

For further information on the development of this new high-throughput tool based on microfluidic real-time PCRs, including sensitivity and specificity tests, as well as the controls employed, please refer to Ref. [41]. Samples with Cycle Threshold (Ct) values below 35, indicating a positive result for infectious agents in the microfluidic real-time PCR assay, underwent further evaluation. To validate and refine results obtained with the BioMark™ system, conventional and nested PCR assays were employed. Specifically, a subset of representative positive samples underwent nested PCRs using different primers for each microorganism compared to the microfluidic real-time PCR. These primers followed published thermal protocols. Additionally, the nested PCRs were used to amplify gene fragments, providing supplementary molecular information about the identified agents. This approach aimed to enhance the robustness and reliability of the results obtained through a comprehensive evaluation process.

#### 2.4.4 Molecular confirmation of *Bartonella* infection in mites

Efforts were made to confirm the presence of *Bartonella* by amplifying of the citrate synthase (*gltA*) gene, using bart781 (5'-GGG GAC CAG CTC ATG GTG G-3') and bart1137 (5'-AAT GCA AAA AGA ACA GTA AAC A-3') primers [42]. DNA electrophoresis revealed positive signals for the intended sequence, indicating the correct amplicon size of 380–400 base pairs (bp) in the four mite pool samples. Sequencing was commissioned to Eurofins MWG Operon (Ebersberg, Germany).

### 2.5 Molecular identification of mites

To determine the species identity, lineage, and genetic diversity of the mites collected in this study, we amplified a 681 bp fragment of the cytochrome *c* oxidase I (*cox1*) from the mitochondrial DNA in two of the four mite pools. The amplification was achieved using COI1Fyuw114 (5′-AGATCTTTAATTGAAGGGGG-3′) and COI1Ryuw114 (5′- AAGATCAAAGAATCGGTGG-3′) primers, corresponding to nucleotide positions 61 to 742 [43]. Subsequently, the amplicons were sent to Eurofins MWG Operon (Ebersberg, Germany) for Sanger sequencing, and the resulting sequences were assembled using BioEdit software (Ibis Biosciences, Carlsbad). The nucleotide sequence data reported in this study have been deposited in the GenBank, EMBL, and DDBJ databases under the accession numbers OR126889, and OR126892.

### 2.6 Phylogenetic and haplotype analysis of mite *cox1* sequences

For analysis of the nucleotide sequences, we utilized the GenBank database via the National Center for Biotechnology Information (NCBI; Bethesda, MD), employing the Basic Local Alignment Sequence Tool (BLAST) search engine (www.ncbi.nlm.nih.gov/blast, accessed on April 5, 2023). Using this search tool, the number of searched sequences was limited to 1000 showing the highest similarity to *cox1* sequences from the current study. Only sequences with the full genus and species name of the parasite were included in further analyses. Next, an initial multiple sequence alignment was performed using the online tool MAFFT v 7.0 [44], and redundant sequences (identical, showing 100 % similarity) were removed from further analyses. The obtained result was saved as FASTA format and analysed using MEGA 11 software [18,45], including alignment based on the MUSCLE algorithm [46]. For tree construction using the Maximum Likelihood method, we selected the Tamura 3-parameter model (T92) based on the lowest Bayesian Information Criterion (BIC) and Corrected Akaike Information Criterion (AICc) available in MEGA 11 [45].

To group the analysed sequences into haplotypes, we used DnaSP software (Universitat de Barcelona, Spain, http://www.ub.edu/dnasp). In addition, the Median Joining Network method available in POPArt software (University of Otago Popart, https://popart.maths.otago.ac.nz), was employed to visualize genealogical relationships at the intraspecific level among studied sequences (obtained in the current study and derived from BLAST).

Based on the phylogenetic tree topology and literature references [28,47,48], the analysed sequences were classified into clades, specifically *D. gallinae* sensu stricto (s.s.), including haplogroups A, B, C; and *D. gallinae* L1. To assess nucleotide divergence between the sequences obtained in this study and those grouped in the *D. gallinae* s.s. and L1 clades [28], we performed calculations using DnaSP software (Universitat de Barcelona, Spain, http://www.ub.edu/dnasp). Furthermore, we tested the statistical significance of the evolutionary rate equality between our sequences and those available in the BLAST database recognized as *D. gallinae* L1. We conducted this test using Tajima's relative rate test under the null hypothesis of equal rates between lineages. All statistical calculations were performed using MEGA 11 [49].

### 2.7 Serological analysis in patients

#### 2.7.1 Testing for IgM and IgG antibodies reactive with *Bartonella* spp. and *Ehrlichia* spp. in human serum samples

The presence of anti-*Ehrlichia* spp. IgM and IgG was assessed using an indirect immunofluorescent test (IFAT). Slides coated with *Ehrlichia canis*-infected cells (MegaFLUO® Ehrlichia canis; Megacor Diagnostik GmbH, Gemeinde Hörbranz, Austria; Cat. no 715K10FK1) were utilized for this purpose. Test sera were diluted with phosphate-buffered saline (PBS) at a ratio of 1:50, as described by Brouqui et al. [50], and then incubated with the antigen-coated slides for 45 min in a humidified chamber at 37 °C. After two PBS washes, goat anti-human IgM and goat anti-human IgG labeled with the green fluorescent dye CF™488A (Sigma-Aldrich, Saint Louis, MO, USA; Cat. SAB4600041 and SAB3701362) were added as secondary antibodies at a working dilution of 1:20 (diluted in PBS). To achieve a counterstain concentration of 0.5 %, Evans blue was added to the final mixture. The incubation with the secondary antibodies took place for 45 min in a humidified chamber at 37 °C. Subsequently, the slides were washed twice with PBS (pH 7.2, Thermo Scientific, Waltham, USA). To confirm the presence of *E. canis* antigens on the slides, a positive control (i.e., anti-*E. canis* serum labeled with fluorescein isothiocyanate (FITC)), provided with the MegaFLUO® *Ehrlichia canis* kit, was added to each slide. Furthermore, a negative control (i.e., *E. canis* antigen directly exposed to secondary antibodies without primary antibodies) was added to ensure the absence of non-specific reactions by the secondary antibody.

For the detection of anti-*Bartonella* IgM and IgG, an IFAT was conducted using slides coated with cells infected with *Bartonella henselae* and *Bartonella quintana* (Vircell s.l., Granada, Spain; Cat. No PBAHQG and PBAHQM), following the manufacturer's instructions, where cut-off titres are defined as 1:2 for IgM and 1:64 for IgG.

The fluorescence reactions were visualized using a CELENA® S Digital Imaging System (Logos Biosystems, Gyeonggi-do, South Korea) with a GFP Long Pass filter (Ex470/40, Em500lp).

#### 2.7.2 Testing for IgE antibodies against *D. gallinae* proteins in human serum samples

##### 2.7.2.1 Origin of *D. gallinae* used as antigens

*Dermanyssus gallinae* mites were collected as previously described [21]. Briefly, mite collection involved brushing the interior parts of egg-laying hens’ cages at the International Poultry Testing Station Ústrašice (MTD Ústrašice, Czech Republic). Less than 4 h after collection, mites were briefly anaesthetised with CO_2_ using a Flystuff benchtop flowbuddy complete with standard flypad (Genesee Scientific, USA) and sorted by developmental stages under a binocular microscope according to Di Palma et al. [39]. Three sample replications were prepared, each containing approximately 160 freshly fed individuals with an equal distribution of each feeding stage (i.e., larvae, nymphal stages including protonymphs and deutonymphs, and adults). The individual samples were then washed with 4 × 500 μl sterile PBS using NucleoSpin® Filters (violet rings) from the Total RNA isolation NucleoSpin® RNAII kit (Macherey-Nagel, Germany) with 500×*g* centrifugation after each wash.

##### 2.7.2.2 Preparation of mite soluble protein extracts

*Dermanyssus gallinae* pools were processed by crushing in 300ul of phosphate buffer saline (NaCl 137 mM, KCl 2.7 mM, Na_2_HPO_4_ 10 mM, KH_2_PO_4_ 1.8 mM, pH 7.4) with 1 % Triton (Sigma-Aldrich, Saint Louis, MO, USA) and six steel balls using the homogenizer Precellys®24 Dual (Bertin, France) at 5500 rpm for 30 s, 3 times. Proteins were quantified using the BCA Protein Assay (ThermoFisher, Waltham, MA, USA) with BSA as standard.

##### 2.7.2.3 Detection of specific anti-*D. gallinae* IgE

To coat the ELISA plates, soluble protein extracts from *D. gallinae* mites were used as mite antigens. Each well of the plates was coated with 50 ng of mite proteins in carbonate/bicarbonate buffer and incubated overnight at 4 °C. After four washes with 200 μl of PBST (PBS containing 0.05 % Tween 20), blocking was performed by adding 100 μl per well of 1 % human serum albumin (HSA) in PBS, followed by incubation at room temperature for 1 h. Another four washes with PBST were carried out before adding serum samples from all patients (including a paired serum sample from Patient 1) and control sera at a 1:50 dilution in PBS. The plates were incubated for 1 h at 37 °C and then washed four times with PBST.

Next, 100 μl per well of goat anti-human immunoglobulin-peroxidase IgE (ɛ-chain specific) at a 1:1000 dilution in blocking buffer (1 % HSA in PBS, supplemented with 0.05 % Tween 20) was added (Sigma-Aldrich, Saint Louis, MO, USA). The plates were incubated for 1 h at 37 °C and washed four times with PBST. The reaction was visualized by adding 100 μl of 3,3′,5,5′-tetramethylbenzidine (Promega, Madison, WI, USA) and incubating in the dark at room temperature for 20 min. The reaction was stopped by adding 50 μl of sulfuric acid, and the optical density (O.D.) was measured at 450 nm using an ELX800 ELISA reader (BioTek, VT, USA).

To analyze the results, the average value of the blanks (wells without mite protein coating; *n* = 4) was subtracted from all readings, and the average of three technical replicates for each sample was used. Normalized values were compared between patients and the control serum (provided by blood donors) using Student's *t*-test with unequal variance (α = 0.05; *n* = 2 biological replicates).

## 3 Results

### 3.1 Mite identification and most-recent-blood meal analysis

**Fig. 2:**
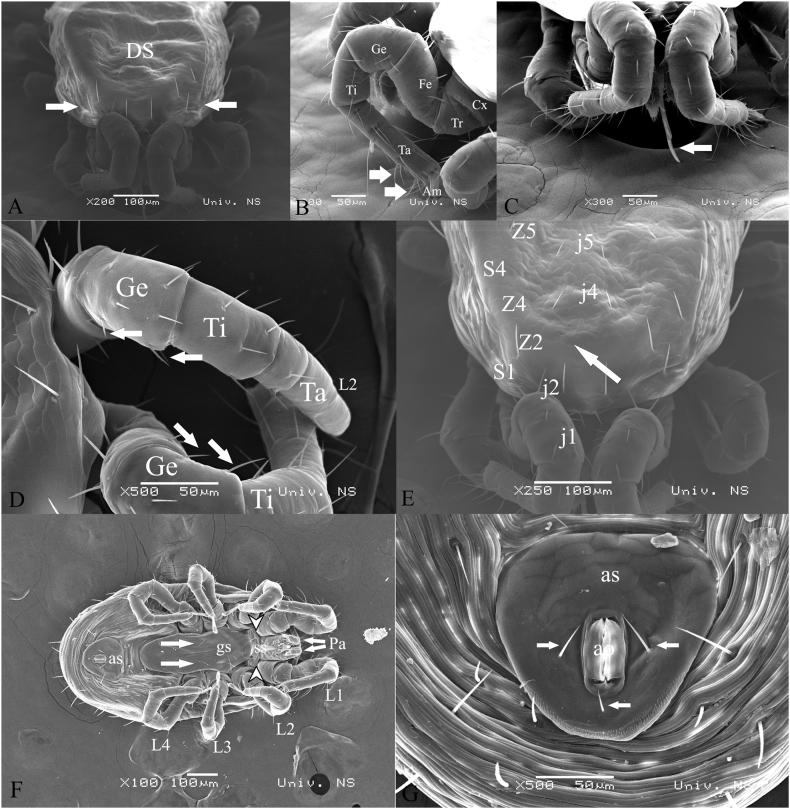
**Scanning electron microscope images depicting the dorsal and ventral sides of a female *Dermanyssus gallinae*.** (A) Dorsal shield with prominent shoulder (arrows). (B) Different size and shape of trichoid sensory receptors (*Sensilla chaetica*) on the first pair of legs (arrows). (C) Anterior region of the body. Second cheliceral articles are elongated and exceeding the basal segment in length (arrow). (D) Genu II with posterolateral seta (arrows pointing to the seta). (E) Dorsal shield with two pars of seta (j1 and j2) with absence of j3 (arrow). (F) Sternal shiled is winder than longer and carries 1–2 pairs bearing 1–2 pairs of sternal setae (white arrowheads). Genitoventral (epigynal) shield is posteriorly rounded. (G) Anal shield is in triangular shape with distinguishable anal orifice surrounded by three setae (arrows). DS-Dorsal shield; Cx-coxa; Tr-trochanter; Fe-femur; Ge-genu; Ti-tibia; Ta-tarsus; Am-ambulacrum; L1-L4-leg; Pa-pedipalp; ss-sternal shield; gs-genitoventral shield; as-anal shield; ao-anal opening.

Based on the morphology of the dorsal and ventral sides (Fig. 2A–G), all the collected mites (*n* = 98) were identified as *Dermanyssus gallinae*. Furthermore, upon closer examination, it was observed that all mites displayed detectable blood in three pairs of caeca and the hindgut. This corresponded to a Score of 2, indicating a two-day period of digestion. Hence, it can be concluded that all mites had most recently fed on blood on the same day that our patients reported the infestation, which was June 13th.

### 3.2 Identification of pathogens in mites, platelets, and blood samples

Molecular diagnosis was conducted on both blood samples and *D. gallinae* samples using high-throughput microfluidic real-time PCR. To detect pathogen DNA, *D. gallinae* specimens were grouped into four pools. Pathogens were exclusively detected in the *D. gallinae* pools, with a 100 % detection rate (4/4). Conversely, none of the collected blood samples from poultry (0 %, 0/4) or family members (0 %, 0/3) tested positive for the presence of tick-borne pathogens (TBPs), including platelet fractions (0 %, 0/3). Additionally, the capillary blood sample collected from the site where *D. gallinae* was biting also tested negative for all the pathogens (0 %, 0/1).

Regarding the *D. gallinae* samples, all four pools tested positive for *Bartonella* spp. (100 %, 4/4). Two pools were positive for *Ehrlichia* spp. (50 %, 2/4), while Apicomplexa and *Theileria* spp. were each detected once (25 %, 1/4) (Table 1). Three pools showed the presence of more than one pathogen. Specifically, we found that two pools tested positive for *Ehrlichia* spp. and *Bartonella* spp., while another pool exhibited the presence of *Bartonella* spp. and Apicomplexa and *Theileria* spp. Efforts to sequence gene fragments from *Bartonella* citrate synthase (gltA) were unsuccessful.

**Table 1:** Summary of clinical, xenodiagnostic, and laboratory findings in patients and poultry following *D. gallinae* infestation case.

Parameters	Humans			Animals			
	Patient 1	Patient 2	Patient 3	Chicken 1	Chicken 2	Chicken 3	Turkey
General data							
Signs of illness	Dermatitis	Not observed	Not observed	Not observed	Not observed	Not observed	Not observed
**Signs and symptoms**							
Fever	–	/	/	/	/	/	/
Headache	–	/	/	/	/	/	/
Myalgia	–	/	/	/	/	/	/
Eschar	–	/	/	/	/	/	/
Enlarged lymph nodes	–	/	/	/	/	/	/
Non-expanding local redness	+	/	/	/	/	/	/
Expanding local redness	–	/	/	/	/	/	/
Itching sensation at lesion site	–	/	/	/	/	/	/
**Serological findings in serum collected one day after infestation incident**							
Anti-*Bartonella* IgM	–	–	–	N/A			
Anti-*Bartonella* IgG	–	–	–				
Anti-*Ehrlichia* IgM	–	–	–				
Anti- *Ehrlichia* IgG	–	–	–				
Anti-*D. gallinae* IgE	+ (0.097)*	+ (0.028)*	+ (0.022)*				
**Serological findings in serum collected four weeks after infestation incident**							
Anti-*Bartonella* IgM	–	N/A		N/A			
anti-*Bartonella* IgG (reactive titer)	+ (1:1024)*						
Anti-*Ehrlichia* IgM	–						
Anti- *Ehrlichia* IgG	–						
Anti-*D. gallinae* IgE (O.D. 450 nm)	+ (0.071)*						
**Detection of TBPs**							
Complete blood	–	–	–	–	–	–	–
Capillary blood from lesion	–	/	/	/	/	/	/
Platelets	–	–	–	–	–	–	–
*D. gallinae* pool 1	*Bartonella* spp. and *Ehrlichia* spp.	N/A					
*D. gallinae* pool 2	*Bartonella* spp. and *Ehrlichia* spp.						
*D. gallinae* pool 3	*Bartonella* spp. and *Theileria* spp. and Apicomplexa						
*D. gallinae* pool 4	*Bartonella* spp.						

Not analysed (N/A).

Absent or not measured are denoted with (/).

Positive (+).

Negative (−).

O.D. values measured at 450 nm (*).

### 3.3 Detection of antibodies against mite and pathogens proteins in human serum samples

**Fig. 3:**
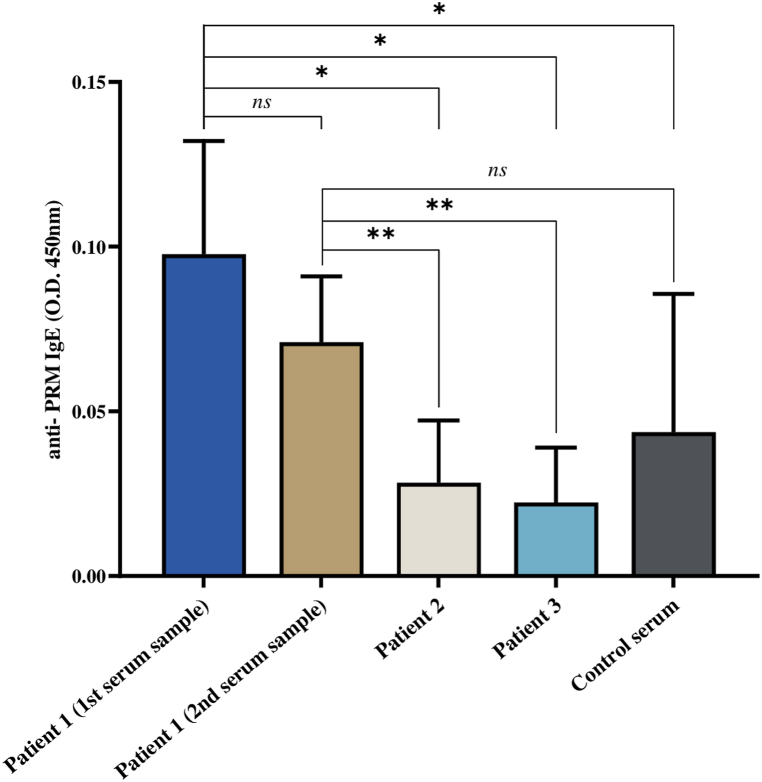
**Detection of IgE antibodies to *Dermanyssus gallinae* proteins in family members and control subjects.** Antibody titres were determined as O.D. at 450 nm and presented as mean (±SD; N = 3 technical replicates). We found significant difference in seroreactivity of 1st and convalescent serum of Patient 1 (i.e., the father) when compared to all other family members and control sample. Antibody titers from patient were compared by Student's *t*-test with unequal variance (*p < 0.05, **p < 0.001, *ns* – not significant, *n* = 3 biological replicates).

Upon analysing serum samples from both patients and control individuals, we observed elevated IgE seroreactivity against mite proteins exclusively in the family member displaying mite bite lesions (Patient 1). Over a two-week period, the IgE levels in this patient decreased but they still remained significantly elevated compared to the IgE levels observed in other family members (Fig. 3).

Furthermore, paired serum samples collected from Patient 1 underwent an IFAT, revealing no IgM or IgG seroreactivity against *Ehrlichia canis* antigens. In contrast, serum sample from Patient 1 a day after *D. gallinae* infestation tested negative for anti-*B. henselae* IgM and IgG, while serum acquired two weeks later from the same patient showed seroreactivity with a titre of 1:1024 for IgG (cut-off 1:64), while remained seronegative for IgM (cut-off 1:2) (Table 1).

### 3.4 Molecular and phylogenetic analyses of *D. gallinae*

**Fig. 4:**
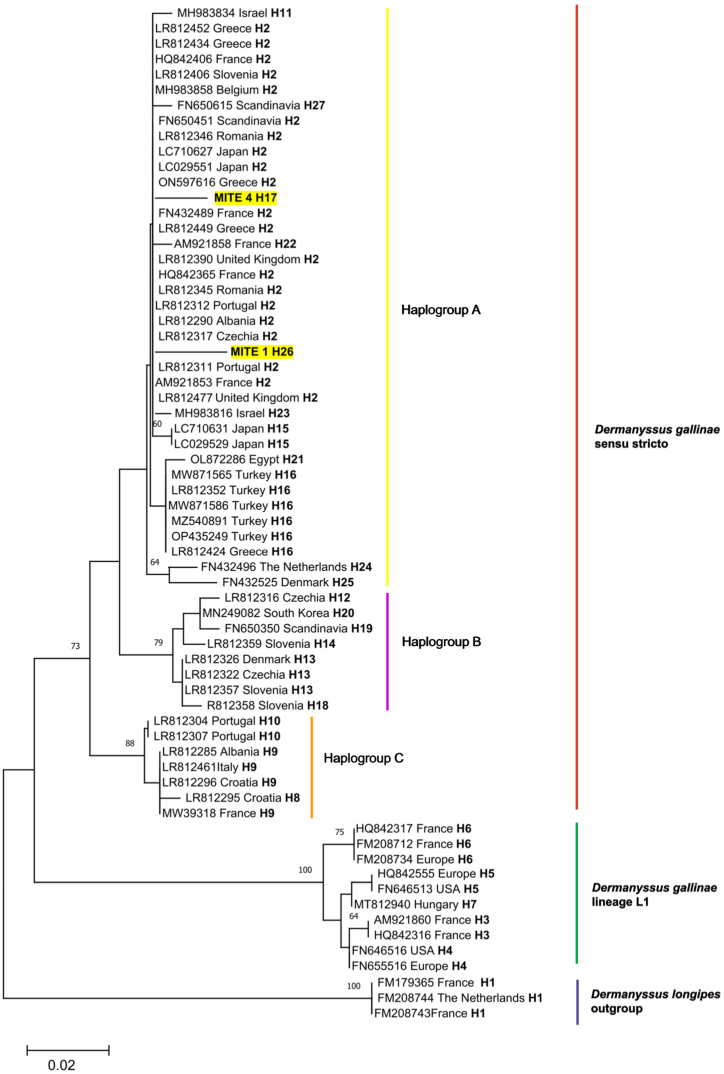
**Phylogeny and haplotype diversity of *Dermanyssus gallinae* inferred from COI mtDNA**. The evolutionary history was inferred by using the Maximum Likelihood method and the Tamura 3-parameter model. The analysis contains sequences identified in current study (**bold**) and GenBank sequences. Accessions numbers and of sequences are given. Analysed sequenced were grouped into haplotypes (H). *Dermanyssus gallinae* sensu stricto clade was also divided into haplogroups A, B and C. Bootstrap values are represented as per cent of internal branches (500 replicates), values lower than 60 are hidden. The tree is drawn to scale, with branch lengths measured in the number of substitutions per site. This analysis involved 66 nucleotide sequences. There was a total of 218 positions in the final dataset. *Dermanyssus longipes* was used as outgroup. Sequences obtained in the current study are highlighted yellow. (For interpretation of the references to colour in this figure legend, the reader is referred to the Web version of this article.)

In this study, two mite pool samples, Mite 1 (OR126889) and Mite 4 (OR126892), were sequenced. These samples were found to cluster together with sequences previously reported from European countries such as Greece (LR812434), Romania (LR812346), and France (HQ842365), as well as from the Far East, specifically Japan (LC029551) (Fig. 4).

**Fig. 5:**
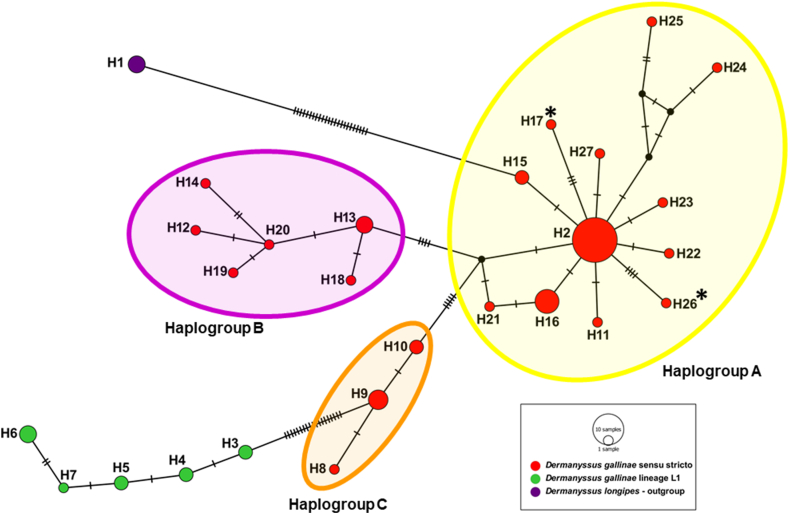
**Median-joining network of haplotypes of *Dermanyssus gallinae* inferred from COI mtDNA.** This figure shows visualisation of genetic relationships among analysed sequences of *Dermanyssus gallinae*, corresponding to records showed in Fig. 4. Haplotypes identified in the current study (H17, H26) fits to *D. gallinae* sensu stricto, haplogroup A. The proportional size of nodes indicates the frequency of haplotypes. The coloured circles match to the coloured clades and haplogroups in the phylogenetic tree (Fig. 4). Small black dots represent inferred haplotypes, while diagonal lines refer to number of mutations between haplotypes. Sequences obtained in the current study are marked with asterix.

Phylogenetic analysis revealed the presence of 26 haplotypes of *D. gallinae*. Among these, 21 haplotypes formed a clade identified as *D. gallinae* sensu stricto (s.s.), while the remaining 5 haplotypes clustered in a distinct clade known as *D. gallinae* L1 (Fig. 5). Among sequences recognized as *D. gallinae* s.s., we confirmed the presence of three haplogroups, i.e., A, B and C (Fig. 4, Fig. 5). The majority of the sequences used for phylogenetic analysis displayed similarity to haplotype H2 (Fig. 5). However, the samples from the current study formed two separate genotypes, namely H17 and H26, respectively (Fig. 4).

When examining the nucleotide divergence, it was observed that sequences Mite 1 (OR126889) exhibited lower divergence with other sequences in clade *D. gallinae* s.s. compared to those in clade L1 (K = 0.0287 and K = 0.1470, respectively). Similarly, Mite 4 displayed lower divergence with sequences in clade *D. gallinae* s.s. compared to those in clade L1 (K = 0.024 and K = 0.1304, respectively). Furthermore, statistical calculations confirmed the rejection of the null hypothesis of equal rates between sequence Mite 1 (χ2 = 39.47, *p* < 0.01), Mite 4 (χ2 = 58.70, *p* < 0.01), and sequences belonging to the L1 lineage. Consequently, the sequences obtained in the current study should be classified as *D. gallinae* s.s. (Fig. 4).

## 4 Discussion

While gamasoidosis caused by *D. gallinae* is reported worldwide, these ectoparasites are generally neglected as potential source of nuisance and infection in humans and cohabiting animals [24,27,[31], [32], [33],^51^]. Human infestations with *D. gallinae* are not commonly reported in European countries, including Serbia [52], possibly due to misdiagnosis and lack of awareness within medical professionals. Differentiating between gamasoidosis and other conditions that cause pruritic skin eruptions is challenging without prior knowledge of *D. gallinae*'s life cycle and distribution [23,31,33,53]. Therefore, it is not surprising that the patients suffering from gamasoidosis caused by *D. gallinae* were previously misdiagnosed as delusional parasitosis [32], Pediculosis corporis [54], scalp pruritus [30], or pseudoscabies [33]. This report presents a holistic approach to managing a household infestation of *D. gallinae*. It includes testing all possible components of a localized epidemiological chain, clinical observation, and examination of pathogen exposure in patients. The specific position of *D. gallinae* as a parasite capable of causing significant economic loss, infesting a wide range of hosts, and damaging their health requires a multidisciplinary approach involving medical and veterinary professionals. This approach should also include urban planners who can develop concrete plans to protect health in urban environments, including hospitals [25,26,55].

In this study, the investigated sequences were molecularly identified as *D. gallinae* s.s., similar to other records reported from Europe and Asia (Fig. 4). Despite molecular differences and high host specificity, both cryptic species, namely *D. gallinae* s.s. and *D. gallinae* lineage L1, have been confirmed to occasionally infest humans [24,28,47]. Previously published reports indicate that poultry keepers and farmers are at the highest risk of *Dermanyssus* mite infestation due to their proximity to birds, henhouses, or nests [24,56]. The *D. gallinae* s.s. infestation confirmed in this study aligns with published data indicating an association of this species with poultry, while the synanthropic *D. gallinae* L1 is linked to pigeons [47].

Although the family members described here kept poultry in a henhouse, the anamnestic data and household organization suggest that the *D. gallinae* s.s. infestation described here is likely a consequence of the proximity of wild and synanthropic bird nests to their common walking route. This infestation also coincided with the time of the year when bird offspring were flying away from their nests, leaving the mites without a host for a blood meal.

*Dermanyssus gallinae* s.s. collected from the patient's shirt tested positive for several tick-borne pathogens, including *Ehrlichia* spp., *Bartonella* spp., and *Theileria* spp. However, none of these pathogens were found in the blood of the poultry kept in the henhouse or in the blood or platelets of the family members. Assuming that the examined mites were indeed feeding on wild or synanthropic birds, there is a possibility that infection with pathogens such as *Ehrlichia* spp. and *Theileria* spp., occurred during co-feeding with arthropods that are competent vectors for those pathogens (e.g., *Ixodes ricinus*) [57,58], through saliva-assisted transmission [59]. Accidental infection via co-feeding phenomena may also explain the presence of other TBPs (i.e., *Coxiella burnetii* and *Borrelia burgdorferi*) previously detected in *D. gallinae* [60].

Some of the microorganisms detected here may be endosymbionts, such as *Bartonella*-like bacteria, which covered between 30 % and 70 % of the sequences in the microbiome of *D. gallinae* collected from poultry houses in Czechia [7,20]. However, it has been previously demonstrated that *Dermanyssus* is capable of carrying and transmitting pathogenic *Bartonella* species to humans, such as *Bartonella quintana* [22]. The presence of *Bartonella*-like bacteria [20] and *Bartonella* pathogens [22] in *D. gallinae* may reflect the microbial evolution along the mutualist–pathogen continuum [61], as described in tick-associated microbes [62,63]. Microorganisms, including those found in *D. gallinae*, are highly adaptable and can rapidly evolve in response to new environments [61]. Mutualistic relationships can break down into parasitism, albeit infrequently in nature [61]. This breakdown may occur due to the spread of cheater symbionts exploiting host benefits without reciprocating [64,65]. The predominant presence of *Bartonella*-like bacteria in *D. gallinae* suggests that these microorganisms may be undergoing dynamic shifts along the mutualist–pathogen continuum, with implications for the nature of their interactions with the mite host and disease ecology. Further research in this area could provide valuable insights into the evolutionary processes shaping these symbiotic associations.

In the case of the gamasoidosis described here, the patient did not develop any signs of bartonellosis, anaplasmosis, or theileriosis. Additionally, we found no evidence of patient exposure to *Ehrlichia* spp. since the patient was seronegative for both anti-*Ehrlichia* IgM and IgG. On the other hand, seroconversion against *B. henselae* observed in Patient 1 is possibly a consequence of exposure to *Bartonella* spp. via *D. gallinae* bites.

Besides the risk of pathogen transmission, *D. gallinae* infestation may trigger hypersensitivity reactions in humans and cause sensitization without allergy in dogs [66,67]. The major allergen identified in dogs is the actin-binding protein tropomyosin [68]. Although the IgE response in individuals infested with *D. gallinae* has not been fully investigated, previous studies have shown that tropomyosin in *D. gallinae* has significant homology with tropomyosins present in arthropods such as *Rhipicephalus microplus*, *Haemaphysalis longicornis*, *Ornithonyssus bursa*, and house dust mites [68,69]. Therefore, it is probable that a person allergic to *D. gallinae* bites will develop hypersensitivity to bites of other arthropods with homologous tropomyosin due to antibody cross-reaction. In this case, we have demonstrated that the patient with clinical manifestations of gamasoidosis had significantly higher levels of anti-*D. gallinae* IgE compared to other family members who did not develop any pruritic lesions. It is important to highlight that anti-*D. gallinae* IgE levels decreased four weeks after infestation but remained elevated compared to other family members. This finding may be important for future studies on the humoral immune response in individuals infested with *D. gallinae* or other ectoparasites with homologous tropomyosin.

## 5 Conclusions

In conclusion, this study identified *D. gallinae* as the causative agent of an infestation in a household. The mites collected from the infested areas had recently fed on blood, confirming their involvement in the reported infestation. The infestation led to clinical manifestations in one family member, characterized by severe itching and skin lesions resembling hives. However, these symptoms resolved within a few weeks without any signs of acute infection. Molecular analysis of the mites revealed the presence of several tick-borne pathogens, including *Bartonella* spp., *Ehrlichia* spp., and *Theileria* spp. These pathogens were exclusively detected in the mites, while no pathogens were found in the blood samples of poultry or other family members. Nevertheless, seroconversion for anti-*Bartonella* IgG was observed in patient who was exposed to *D. gallinae* infestation, suggesting possibility that *D. gallinae* can act as vector for these pathogens. Antibody analysis showed elevated levels of IgE against mite proteins in the infested family member, indicating an allergic response to the mite bites. The household environment included a chicken coop and nearby bird nests, suggesting a potential source of infestation. Molecular and phylogenetic analysis confirmed the identification of *D. gallinae* samples and revealed two distinct genotypes within the *D. gallinae* species. These findings highlight the potential health risks associated with *D. gallinae* infestations and emphasize the need for proper management and control measures. The study provides valuable insights into the epidemiology, clinical presentation, and pathogen detection associated with *D. gallinae* infestations. Further research is warranted to investigate the exact mechanisms of pathogen transmission by *D. gallinae* and to develop effective strategies for prevention and control of infestations. Our results are highlighting the importance of conducting further research and increasing awareness to ensure timely diagnosis, treatment, and preventive measures, particularly in severe cases or among individuals with heightened sensitivities.

### Data availability statement

The nucleotide sequence data reported in this study have been deposited in the GenBank, EMBL, and DDBJ databases under the accession numbers OR126889, and OR126892.

## Ethics statement

The study received ethical approval (Ethical approval no. 01–39/206/1) from the Комисија за етичност клиничких испитивања Медицинског факултета Нови Сад, Универзитета у Новом Саду, and adhered to the guidelines set forth in the Helsinki Declaration and the Patient Rights Law of the Republic of Serbia. Prior to publication, written informed consent for the publication of this clinical case report was obtained from the patients involved. Furthermore, the handling of household animals and their blood samples followed the guidelines outlined in the EU Directive 2010/63/EU for animal experimentation.

## CRediT authorship contribution statement

**Pavle Banović:** Writing – review & editing, Writing – original draft, Visualization, Methodology, Investigation, Formal analysis, Conceptualization. **Angélique Foucault-Simonin:** Writing – review & editing, Validation, Investigation, Data curation. **Luka Papić:** Writing – review & editing, Resources, Investigation. **Sara Savić:** Writing – review & editing, Resources, Investigation. **Aleksandar Potkonjak:** Writing – review & editing, Resources, Investigation. **Aleksandar Jurišić:** Writing – review & editing, Resources, Investigation. **Marko Radenković:** Writing – review & editing, Visualization, Investigation. **Dragana Mijatović:** Writing – review & editing, Resources, Investigation. **Verica Simin:** Writing – review & editing, Resources, Investigation. **Ivana Bogdan:** Writing – review & editing, Resources, Investigation. **Zbigniew Zając:** Writing – review & editing, Visualization, Methodology, Investigation, Formal analysis. **Joanna Kulisz:** Writing – review & editing, Visualization, Methodology, Formal analysis. **Aneta Woźniak:** Writing – review & editing, Visualization, Methodology, Formal analysis. **David Hartmann:** Writing – review & editing, Methodology, Investigation. **Jan Perner:** Writing – review & editing, Methodology, Investigation. **Alejandra Wu-Chuang:** Writing – review & editing, Data curation. **Lourdes Mateos-Hernandez:** Writing – review & editing, Methodology, Investigation. **Sara Moutailler:** Writing – review & editing, Supervision, Resources, Methodology. **Alejandro Cabezas-Cruz:** Writing – review & editing, Writing – original draft, Supervision, Resources, Conceptualization.

## Declaration of competing interest

The authors declare that they do not have any conflicts of interests associated with the research presented here.
