# Supplementary material for: One health approach to study human health risks associated with Dermanyssus gallinae mites
Source: Heliyon. 2024 May 1;10(9):e30539. doi: 10.1016/j.heliyon.2024.e30539 (PMC11089355; doi:10.1016/j.heliyon.2024.e30539)
Supplement: Multimedia component 1 [file mmc1.doc]

**Supplementary Table S1. Primers and probes used in the study.**

| **Organism** | **Targeted gene** | **Primers (F and R - 5'-3') and Probes (P)** | **Product length (bp)** | **Reference** |
| --- | --- | --- | --- | --- |
| *Borrelia burgdorferi* sensu stricto | *rpoB* | F-GCTTACTCACAAAAGGCGTCTT | 83 | Michelet et al. 2014 |
|  |  | R-GCACATCTCTTACTTCAAATCCT |  |  |
|  |  | P-AATGCTCTTGGACCAGGAGGACTTTCA |  |  |
| *Borrelia garinii* | *rpoB* | F-TGGCCGAACTTACCCACAAAA | 88 | Michelet et al. 2014 |
|  |  | R-ACATCTCTTACTTCAAATCCTGC |  |  |
|  |  | P-TCTATCTCTTGAAAGTCCCCCTGGTCC |  |  |
| *Borrelia afzelii* | *fla* | F-GGAGCAAATCAAGATGAAGCAAT | 116 | Michelet et al. 2014 |
|  |  | R-TGAGCACCCTCTTGAACAGG |  |  |
|  |  | P-TGCAGCCTGAGCAGCTTGAGCTCC |  |  |
| *Borrelia valaisiana* | *ospA* | F-ACTCACAAATGACAGATGCTGAA | 135 | Michelet et al. 2014 |
|  |  | R-GCTTGCTTAAAGTAACAGTACCT |  |  |
|  |  | P-TCCGCCTACAAGATTTCCTGGAAGCTT |  |  |
| *Borrelia lusitaniae* | *rpoB* | F-CGAACTTACTCATAAAAGGCGTC | 87 | Michelet et al. 2014 |
|  |  | R-TGGACGTCTCTTACTTCAAATCC |  |  |
|  |  | P-TTAATGCTCTCGGGCCTGGGGGACT |  |  |
| *Borrelia spielmanii* | *fla* | F-ATCTATTTTCTGGTGAGGGAGC | 71 | Michelet et al. 2014 |
|  |  | R-TCCTTCTTGTTGAGCACCTTC |  |  |
|  |  | P-TTGAACAGGCGCAGTCTGAGCAGCTT |  |  |
| *Borrelia bissetti* | *rpoB* | F-GCAACCAGTCAGCTTTCACAG | 118 | Michelet et al. 2014 |
|  |  | R-CAAATCCTGCCCTATCCCTTG |  |  |
|  |  | P-AAAGTCCTCCCGGCCCAAGAGCATTAA |  |  |
| *Borrelia myamotoi* | *glpQ* | F-CACGACCCAGAAATTGACACA | 94 | Michelet et al. 2014 |
|  |  | R-GTGTGAAGTCAGTGGCGTAAT |  |  |
|  |  | P-TCGTCCGTTTTCTCTAGCTCGATTGGG |  |  |
| *Borrelia* spp. | *23S rRNA* | F-GAGTCTTAAAAGGGCGATTTAGT | 73 | Michelet et al. 2014 |
|  |  | R-CTTCAGCCTGGCCATAAATAG |  |  |
|  |  | P-AGATGTGGTAGACCCGAAGCCGAGT |  |  |
| *Anaplasma marginale* | *msp1* | F-CAGGCTTCAAGCGTACAGTG | 85 | Michelet et al. 2014 |
|  |  | R-GATATCTGTGCCTGGCCTTC |  |  |
|  |  | P-ATGAAAGCCTGGAGATGTTAGACCGAG |  |  |
| *Anaplasma platys* | *groEL* | F-TTCTGCCGATCCTTGAAAACG | 75 | Michelet et al. 2014 |
|  |  | R-CTTCTCCTTCTACATCCTCAG |  |  |
|  |  | P-TTGCTAGATCCGGCAGGCCTCTGC |  |  |
| *Amnaplasma phagocytophilum* | *msp2* | F-GCTATGGAAGGCAGTGTTGG | 77 | Michelet et al. 2014 |
|  |  | R-GTCTTGAAGCGCTCGTAACC |  |  |
|  |  | P-AATCTCAAGCTCAACCCTGGCACCAC |  |  |
| *Anaplasma ovis* | *msp4* | F-TCATTCGACATGCGTGAGTCA | 92 | Michelet et al. 2014 |
|  |  | R-TTTGCTGGCGCACTCACATC |  |  |
|  |  | P-AGCAGAGAGACCTCGTATGTTAGAGGC |  |  |
| *Anaplasma centrale* | *groEL* | F-AGCTGCCCTGCTATACACG | 79 | Michelet et al. 2014 |
|  |  | R-GATGTTGATGCCCAATTGCTC |  |  |
|  |  | P-CTTGCATCTCTAGACGAGGTAAAGGGG |  |  |
| *Anaplasma bovis* | *groEL* | F-GGGAGATAGTACACATCCTTG | 73 | Sprong et al. 2019 |
|  |  | R-CTGATAGCTACAGTTAAGCCC |  |  |
|  |  | P-AGGTGCTGTTGGATGTACTGCTGGACC |  |  |
| *Anaplasma* spp. | *16S rRNA* | F-CTTAGGGTTGTAAAACTCTTTCAG | 160 | Gondard et al. 2020 |
|  |  | R-CTTTAACTTACCAAACCGCCTAC |  |  |
|  |  | P-ATGCCCTTTACGCCCAATAATTCCGAACA |  |  |
| *Ehrlichia canis* | *gltA* | F-GACCAAGCAGTTGATAAAGATGG | 136 | Gondard et al. 2020 |
|  |  | R-CACTATAAGACAATCCATGATTAGG |  |  |
|  |  | P-ATTAAAACATCCTAAGATAGCAGTGGCTAAGG |  |  |
| *Ehrlichia/Neoehrlichia* spp. | *16S rRNA* | F-GCAACGCGAAAAACCTTACCA | 98 | Gondard et al. 2020 |
|  |  | R-AGCCATGCAGCACCTGTGT |  |  |
|  |  | P-AAGGTCCAGCCAAACTGACTCTTCCG |  |  |
| *Candidatus* Neoehrlichia mikurensis | *groEL* | F-AGAGACATCATTCGCATTTTGGA | 96 | Michelet et al. 2014 |
|  |  | R-TTCCGGTGTACCATAAGGCTT |  |  |
|  |  | P-AGATGCTGTTGGATGTACTGCTGGACC |  |  |
| *Rickettsia conorii* | *23S-5S ITS* | F-CTCACAAAGTTATCAGGTTAAATAG | 118 | Michelet et al. 2014 |
|  |  | R-CGATACTCAGCAAAATAATTCTCG |  |  |
|  |  | P-CTGGATATCGTGGCAGGGCTACAGTAT |  |  |
| *Rickettsia slovaca* | *23S-5S ITS* | F-GTATCTACTCACAAAGTTATCAGG | 138 | Michelet et al. 2014 |
|  |  | R-CTTAACTTTTACTACAATACTCAGC |  |  |
|  |  | P-TAATTTTCGCTGGATATCGTGGCAGGG |  |  |
| *Rickettsia massiliae* | *23S-5S ITS* | F-GTTATTGCATCACTAATGTTATACTG | 128 | Michelet et al. 2014 |
|  |  | R-GTTAATGTTGTTGCACGACTCAA |  |  |
|  |  | P-TAGCCCCGCCACGATATCTAGCAAAAA |  |  |
| *Rickettsia helvetica* | *23S-5S ITS* | F-AGAACCGTAGCGTACACTTAG | 79 | Michelet et al. 2014 |
|  |  | R-GAAAACCCTACTTCTAGGGGT |  |  |
|  |  | P-TACGTGAGGATTTGAGTACCGGATCGA |  |  |
| *Rickettsia aeshlimannii* | *23S-5S ITS* | F-CTCACAAAGTTATCAGGTTAAATAG | 134 | Sprong et al.2019 |
|  |  | R-CTTAACTTTTACTACGATACTTAGCA |  |  |
|  |  | P-TAATTTTTGCTGGATATCGTGGCGGGG |  |  |
| *Rickettsia felis* | *orfB* | F-ACCCTTTTCGTAACGCTTTGC | 163 | Gondard et al. 2020 |
|  |  | R-TATACTTAATGCTGGGCTAAACC |  |  |
|  |  | P-AGGGAAACCTGGACTCCATATTCAAAAGAG |  |  |
| *Rickettsia* spp. | *gltA* | F-GTCGCAAATGTTCACGGTACTT | 145 | Michelet et al. 2014 |
|  |  | R-TCTTCGTGCATTTCTTTCCATTG |  |  |
|  |  | P-TGCAATAGCAAGAACCGTAGGCTGGATG |  |  |
| *Bartonella henselae* | *pap31* | F-CCGCTGATCGCATTATGCCT | 107 | Michelet et al. 2014 |
|  |  | R-AGCGATTTCTGCATCATCTGCT |  |  |
|  |  | P-ATGTTGCTGGTGGTGTTTCCTATGCAC |  |  |
| *Bartonella* spp. | *ssrA* | F-CGTTATCGGGCTAAATGAGTAG | 118 | Gondard et al. 2020 |
|  |  | R-ACCCCGCTTAAACCTGCGA |  |  |
|  |  | P-TTGCAAATGACAACTATGCGGAAGCACGTC |  |  |
| *Francisella tularensis* | *tul4* | F-ACCCACAAGGAAGTGTAAGATTA | 76 | Michelet et al. 2014 |
|  |  | R-GTAATTGGGAAGCTTGTATCATG |  |  |
|  |  | P-AATGGCAGGCTCCAGAAGGTTCTAAGT |  |  |
| *Francisella-*like endosymbiont | *fop4* | F-GGCAAATCTAGCAGGTCAAGC | 91 | Michelet et al. 2014 |
|  |  | R-CAACACTTGCTTGAACATTTCTAG |  |  |
|  |  | P-AACAGGTGCTTGGGATGTGGGTGGTG |  |  |
| *Coxiella burnetii* | *IS1111* | F-TGGAGGAGCGAACCATTGGT | 86 | Michelet et al. 2014 |
|  |  | R-CATACGGTTTGACGTGCTGC |  |  |
|  |  | P-ATCGGACGTTTATGGGGATGGGTATCC |  |  |
| *Coxiella-*like | *idc* | F-AGGCCCGTCCGTTATTTTACG | 74 | Michelet et al. 2014 |
|  |  | R-CGGAAAATCACCATATTCACCTT |  |  |
|  |  | P-TTCAGGCGTTTTGACCGGGCTTGGC |  |  |
| *Apicomplexa* | *18S* | F-TGAACGAGGAATGCCTAGTATG | 104 | Gondard et al. 2020 |
|  |  | R-CACCGGATCACTCGATCGG |  |  |
|  |  | P-TAGGAGCGACGGGCGGTGTGTAC |  |  |
| *Babesia microti* | *CCTeta* | F-ACAATGGATTTTCCCCAGCAAAA | 145 | Michelet et al. 2014 |
|  |  | R-GCGACATTTCGGCAACTTATATA |  |  |
|  |  | P-TACTCTGGTGCAATGAGCGTATGGGTA |  |  |
| *Babesia canis* (3 subspecies) | *18 rRNA* | F-TGGCCGTTCTTAGTTGGTGG | 104 | Michelet et al. 2014 |
|  |  | R-AGAAGCAACCGGAAACTCAAATA |  |  |
|  |  | P-ACCGGCACTAGTTAGCAGGTTAAGGTC |  |  |
| *Babesia ovis* | *18S rRNA* | F-TCTGTGATGCCCTTAGATGTC | 92 | Michelet et al. 2014 |
|  |  | R-GCTGGTTACCCGCGCCTT |  |  |
|  |  | P-TCGGAGCGGGGTCAACTCGATGCAT |  |  |
| *Babesia bovis* | *CCTeta* | F-GCCAAGTAGTGGTAGACTGTA | 100 | Michelet et al. 2014 |
|  |  | R-GCTCCGTCATTGGTTATGGTA |  |  |
|  |  | P-TAAAGACAACACTGGGTCCGCGTGG |  |  |
| *Babesia caballi* | *Rap1* | F-GTTGTTCGGCTGGGGCATC | 94 | Michelet et al. 2014 |
|  |  | R-CAGGCGACTGACGCTGTGT |  |  |
|  |  | P-TCTGTCCCGATGTCAAGGGGCAGGT |  |  |
| *Babesia venatorum* (sp. EU1) | *18S rRNA* | F-GCGCGCTACACTGATGCATT | 91 | Michelet et al. 2014 |
|  |  | R-CAAAAATCAATCCCCGTCACG |  |  |
|  |  | P-CATCGAGTTTAATCCTGTCCCGAAAGG |  |  |
| *Babesia divergens* | *hsp70* | CTCATTGGTGACGCCGCTA | 83 | Michelet et al. 2014 |
|  |  | R-CTCCTCCCGATAAGCCTCTT |  |  |
|  |  | P-AGAACCAGGAGGCCCGTAACCCAGA |  |  |
| Mycoplasma spp. | 16S (NR_026155.1) | GTGACGGCTAACTATGTGCC | 77 bp | No submission |
|  |  | CGGAATTATTGGGCGTAAAGC |  |  |
|  |  | AGCAGCTGCGGTAATACATAGGTCGC |  |  |
| *Theileria* spp. | *18S rRNA* | GTCAGTTTTTACGACTCCTTCAG | 213 bp | Submission in progress |
|  |  | CCAAAGAATCAAGAAAGAGCTATC |  |  |
|  |  | AATCTGTCAATCCTTCCTTTGTCTGGACC |  |  |
| *Hepatozoon* spp. | *18S rRNA* | F-ATTGGCTTACCGTGGCAGTG | 175 | Gondard et al. 2020 |
|  |  | R-AAAGCATTTTAACTGCCTTGTATTG |  |  |
|  |  | P-ACGGTTAACGGGGGATTAGGGTTCGAT |  |  |
|  |  | R-CTCATGCGGAAATAGCCGTTA |  |  |
|  |  | P-ATAGTCTCGCCAGTATTCGCCACCAATACC |  |  |

**References**

Gondard M, Delannoy S, Pinarello V, Aprelon R, Devillers E, Galon C, Pradel J, Vayssier-Taussat M, Albina E, Moutailler S. Upscaling the Surveillance of Tick-borne Pathogens in the French Caribbean Islands. Pathogens. 2020;9(3):176. doi: 10.3390/pathogens9030176.

Michelet L, Delannoy S, Devillers E, Umhang G, Aspan A, Juremalm M, Chirico J, van der Wal FJ, Sprong H, Boye Pihl TP, Klitgaard K, Bødker R, Fach P, Moutailler S. High-throughput screening of tick-borne pathogens in Europe. Front Cell Infect Microbiol. 2014;4:103. doi: 10.3389/fcimb.2014.00103.

Sprong H, Fonville M, Docters van Leeuwen A, Devillers E, Ibañez-Justicia A, Stroo A, Hansford K, Cull B, Medlock J, Heyman P, Cochez C, Weis L, Silaghi C, Moutailler S. Detection of pathogens in Dermacentor reticulatus in northwestern Europe: evaluation of a high-throughput array. Heliyon. 2019;5(2):e01270. doi: 10.1016/j.heliyon.2019.e01270.
